# Cellobiose-Mediated Gene Expression in *Streptococcus pneumoniae*: A Repressor Function of the Novel GntR-Type Regulator BguR

**DOI:** 10.1371/journal.pone.0057586

**Published:** 2013-02-28

**Authors:** Sulman Shafeeq, Oscar P. Kuipers, Tomas G. Kloosterman

**Affiliations:** Department of Molecular Genetics, Groningen Biomolecular Sciences and Biotechnology Institute, University of Groningen, Groningen, The Netherlands; Instituto Butantan, Brazil

## Abstract

The human pathogen *Streptococcus pneumoniae* has the ability to use the carbon- and energy source cellobiose due to the presence of a cellobiose-utilizing gene cluster (*cel* locus) in its genome. This system is regulated by the cellobiose-dependent transcriptional activator CelR, which has been previously shown to contribute to pneumococcal virulence. To get a broader understanding of the response of *S. pneumoniae* to cellobiose, we compared the pneumococcal transcriptome during growth on glucose as the main carbon source to that with cellobiose as the main carbon source. The expression of various carbon metabolic genes was altered, including a PTS operon (which we here denote as the *bgu* operon) that has high similarity with the *cel* locus. In contrast to the *cel* locus, the *bgu* operon is conserved in all sequenced strains of *S. pneumoniae*, indicating an important physiological function in the lifestyle of pneumococci. We next characterized the transcriptional regulation of the *bgu* operon in more detail. Its expression was increased in the presence of cellobiose, and decreased in the presence of glucose. A novel GntR-type transcriptional regulator (which we here denote as BguR) was shown to act as a transcriptional repressor of the *bgu* operon and its repressive effect was relieved in the presence of cellobiose. BguR-dependent repression was demonstrated to be mediated by a 20-bp DNA operator site (5′-AAAAATGTCTAGACAAATTT-3′) present in P*bguA*, as verified by promoter truncation experiments. In conclusion, we have identified a new cellobiose-responsive PTS operon, together with its transcriptional regulator in *S. pneumoniae*.

## Introduction

*Streptococcus pneumoniae* is a Gram-positive bacterial pathogen in humans that has the ability to colonize the nasopharyngeal cavity of the nose [1]. In favorable environmental conditions it may spread to different parts of the human body to cause serious infections like pneumonia, otitis media, septicemia or meningitis [2], [3] leading to millions of deaths each year, especially in children and the elderly [4]. In order to survive in the different niches in the human host, *S. pneumoniae* must have the ability to adapt to fluctuating levels of nutrients [5], [6], such as the available carbohydrate- and energy sources.

Previous studies have shown that *S. pneumoniae* is able to use an exceptionally broad spectrum of different carbon sources like cellobiose, raffinose, sucrose, galactose, maltose and others [7]–[18]. Cellobiose is a β-glucoside carbohydrate that can also be utilized by *S. pneumoniae* as an energy source alternative to a preferred sugar like glucose [7], [18], although it is not clear whether it encounters this presumed plant-derived carbohydrate in its natural environment. However, the extracellular matrix of mammalian tissues is rich in glycosaminoglycans that contain repeating units of β-linked disaccharides [19]. The degradation of glycosaminoglycans from these mammalian extracellular tissues may release structural analogues of cellobiose [19], [20]. Therefore, a system previously described to function in cellobiose utilization in *S. pneumoniae*, *i.e.* the Cel system [7], [8], may be involved in the acquisition and metabolism of β-glucosides related to cellobiose, which are derived by degradation of the extracellular matrix or other biopolymers in the host [19], [20].

The Cel system (encoded by the *cel* locus) [7] has been shown to be required for *S. pneumoniae* strain R6 to grow on cellobiose as the sole energy source. The *cel* loci of strains R6/D39 consist of seven genes and they are transcribed into two transcriptional units [7], [8]. The genes in the *cel* locus encode a phospho-β-glucosidase (*celA*), a DNA-binding transcriptional activator (*celR*), cellobiose-specific PTS IIBAC components (*celB*, *celC* and *celD*) and two proteins with unknown functions. CelR was found to be responsible for activation of the *cel* locus in *Streptococcus mutans* in the presence of cellobiose [21]. Recently, CelR also has been demonstrated to function as a transcriptional activator of the *cel* locus in *S. pneumoniae* [8]. However, the *cel* locus is not conserved in all pneumococcal strains, as it is absent in 50% of the sequenced *S. pneumoniae* genomes available on the KEGG website, including major multi-drug resistant pneumococcal strains like 19F and 23F. Based on this observation, we hypothesized that *S. pneumoniae* might possess alternative systems that it employs to use β-glucosides like cellobiose.

To get a broader understanding of the response of *S. pneumoniae* to cellobiose, we examined in this study cellobiose-dependent regulation on a transcriptome-wide level and found another operon, homologous to the *cel* locus, which is highly expressed in the presence of cellobiose. This operon encodes a PTS system that was recently shown to contribute to growth on the β-glucosides amygdalin and cellobiose [17]. In subsequent experiments, this operon was found to be regulated by a GntR-type transcriptional repressor (BguR, encoded by the divergently orientated upstream gene) in response to cellobiose and glucose. A conserved operator sequence was found that is necessary for the regulation to take place. The novel PTS operon, which we tentatively name *bguDBC*, did not contribute to growth in medium with cellobiose as the sole carbon source, whereas the *cel* locus did. Therefore, the *cel* locus seems to be the primary transport system for cellobiose in *S. pneumoniae* D39, but the exact function of *bgu* operon, although likely to lie in transport of β-glucosides [17], is still not entirely clear.

## Materials and Methods

### DNA Manipulation, Bacterial Strains and Growth Conditions

Chromosomal DNA of *S. pneumoniae* D39 wild-type [10] was used for PCR amplification. Primers were based on the sequence of the D39 genome [10] and are listed in Table 1.

**Table 1:** List of primers used in this study. Restriction sites are underlined.

Name	Nucleotide Sequence (5′3′)	Restriction site
PbguA-Fr	CGGGATCCCCGCTAGAAGCTGCTCCCCACC	EcoRI
PbguA-Rv	CGGAATTCCTTTTCACGAATCTCATTGT	BamHI
PmalQ-Fr	CGGGAATTCTATGGACGTTTGTGCTTTG	EcoRI
PmalQ-Rv	CGGGATCCGAGATGTGCATCAACACAC	BamHI
PmalP-Fr	CGGGAATTCCCTCTTTAGACAGATTC	EcoRI
PmalP-Rv	CGGGATCCAAGCACCGCAGTGCTC	BamHI
SPD1830-KO-1	GTAAATTCATCACAAGATCC	–
SPD1830-KO-2	TCCTCCTCACTATTTTGATTAGTTTTGTACTCATTTAATCTGG	–
SPD1830-KO-3	CGTTTTAGCGTTTATTTCGTTTAGTCATTACGACATTCCTCCTAGG	–
SPD1830-KO-4	CTGTTTTCATACTCTTTCCC	–
SPD1832-4-KO-1	CTGGATGCCAGACCAATAC	–
SPD1832-4-KO-2	GAGATCTAATCGATGCATGCCAGCAAAGGTGGCAAATTGG	–
SPD1832-4-KO-3	AGTTATCGGCATAATCGTTAGAATTCATCGATCTCTATC	–
SPD1832-4-KO-4	TTCCTGATAGAGTTGTTCAC	–
Spec-R	ACTAAACGAAATAAACGC	–
Spec-F	CTAATCAAAATAGTGAGGAGG	–
Ery-R	TAACGATTATGCCGATAACT	–
Ery-F	GCATGCATCGATTAGATCTC	–
	**Forward primers used with PbguA-Rv (reverse primer) for 5′ subcloning of P** ***bguA***	
PbguA-5.3	GCATGAATTCTAGACAAATTTTAAAATTATG	EcoRI
PbguA-5.4	GCATGAATTCGTCTAGACAAATTTTAAAATTATGC	EcoRI
PbguA-5.5	GCATGAATTCGTATCAACAATTTTTAAAAATG	EcoRI

Bacterial strains and plasmids used in this study are listed in Table 2. All bacterial strains were stored in 10% (v/v) glycerol at −80°C. M17 broth [22], [23] supplemented with 0.5% (w/v) glucose was used for growing *S. pneumonia*e D39 wild-type [10] on blood agar plates supplemented with 1% (v/v) defibrinated sheep blood in micro-aerophilic conditions at 37°C. For selection, media were supplemented with the following concentrations of antibiotics: erythromycin: 0.25 µg ml^−1^, spectinomycin: 150 µg ml^−1^, tetracycline: 2.5 µg ml^−1^ for *S. pneumoniae*; and ampicillin: 100 µg ml^−1^ for *E. coli*.

**Table 2:** List of strains and plasmids used in this study.

Strain/plasmid	Description	Source
***S. pneumoniae***		
D39	Serotype 2 strain. *cps 2*	Laboratory of P. Hermans.
Δ*ccpA*	D39 Δ*ccpA*; Spec^R^	[13]
Δ*celR*	D39 Δ*celR*; Spec^R^	[8]
Δ*bguDBC*	D39 Δ*bguDBC*; Ery^R^	This study
SS300	D39 Δ*bguR*; Spec^R^	This study
SS301	D39 Δ*bgaA*::P*bguA*-*lacZ*; Tet^R^	This study
SS302	SS300 Δ*bgaA*:: P*bguA*-*lacZ*; Tet^R^	This study
SS303	Δ*ccpA* Δ*bgaA*::P*bguA*-*lacZ*; Tet^R^	This study
SS304	Δ*bguDBC* Δ*bgaA*::P*bguA*-*lacZ*; Tet^R^	This study
SS305	D39 Δ*bgaA*::P*bguA-5.3*-*lacZ*; Tet^R^	This study
SS306	D39 Δ*bgaA*::P*bguA-5.4*-*lacZ*; Tet^R^	This study
SS307	D39 Δ*bgaA*::P*bguA-5.5*-*lacZ*; Tet^R^	This study
***E. coli***		
EC1000	Km^R^; MC1000 derivative carrying a single copy of the pWV1 *repA* gene in *glgB*	Laboratory collection
**Plasmids**		
pPP2	Amp^R^ Tet^R^; promoter-less *lacZ*. For replacement of *bgaA* with promoter *lacZ*-fusion. Derivative of pTP1.	[25]
pSS301	pPP2 P*bguA*-*lacZ*	This study
pSS302	pPP2 P*bguA-5.3*-*lacZ*	This study
pSS303	pPP2 P*bguA-5.4*-*lacZ*	This study
pSS304	pPP2 P*bguA-5.5*-*lacZ*	This study

### Construction of Deletion Mutants of *bguR* and *bguDBC*

*bguR* and *bguDBC* deletion mutants were made by allelic replacement with a spectinomycin- and erythromycin-resistance gene, respectively, following the procedure as described before [24]. Briefly, primer pairs SPD1830-KO-1/SPD1830-KO-2, SPD1830-KO-3/SPD1830-KO-4, 1832-4-KO-1/1832-4-KO-2 and 1832-4-KO-3/1832-4-KO-4 were used to generate PCR fragments of the left- and right flanking regions of *bguR* and *bguDBC*, respectively. The spectinomycin-resistance marker was amplified by a PCR on pORI38 with primers Spec-F/Spec-R. The erythromycin-resistance marker was amplified by a PCR on pORI28 with primers Ery-F/Ery-R. Then, the left- and right flanking regions of *bguR* and *bguDBC* were fused to the spectinomycin- and erythromycin-resistance markers, respectively, by means of overlap-extension PCR. The resulting PCR products were transformed to *S. pneumoniae* D39 wild-type and selection of the mutant strains was done with the appropriate antibiotic. Spectinomycin- and erythromycin-resistant clones were further examined for the presence of the *bguR* and *bguDBC* deletion, respectively, by PCR.

### Construction of *lacZ*-fusions and *bguA* Promoter Subclones in pPP2

The pPP2 [25] plasmid was used to construct a vector for an ectopic chromosomal transcriptional *lacZ*-fusion to the promoter of *bguA* in *S. pneumoniae* D39 using the primer pair mentioned in Table 1. The resulting plasmid pSS301 was introduced into D39 wild-type and the *bguR* mutant and integrated in the genome via double crossover in the *bgaA* gene, resulting in strains SS301 and SS302. The P*bguA-lacZ* was also introduced into the Δ*ccpA* strain that was published before [13] and in the Δ*bguDBC*, resulting in strain SS303 and SS304, respectively.

The following promoter subclones of P*bguA* were made in pPP2 (primer pairs are mentioned in Table 1): P*bguA-5.3* (truncated 15 bases upstream of the proposed BguR operator site), P*bguA-5.4* (first 6 bases of the BguR operator site deleted) and P*bguA-5.5* (half of the BguR operator site deleted, but keeping the -35 site intact). This resulted in plasmids pSS302-04. These constructs were introduced into D39 wild-type as described above, resulting in strains SS305-07. All plasmid constructs were checked by sequencing.

### Enzyme Assays

Specific β-galactosidase assays were performed as described before [26]. Cells were grown in M17 in the presence of the appropriate carbon source (exact concentrations are mentioned in the Results section) and harvested in the mid-exponential phase of growth.

### DNA Microarray Analyses

**Figure 1:**
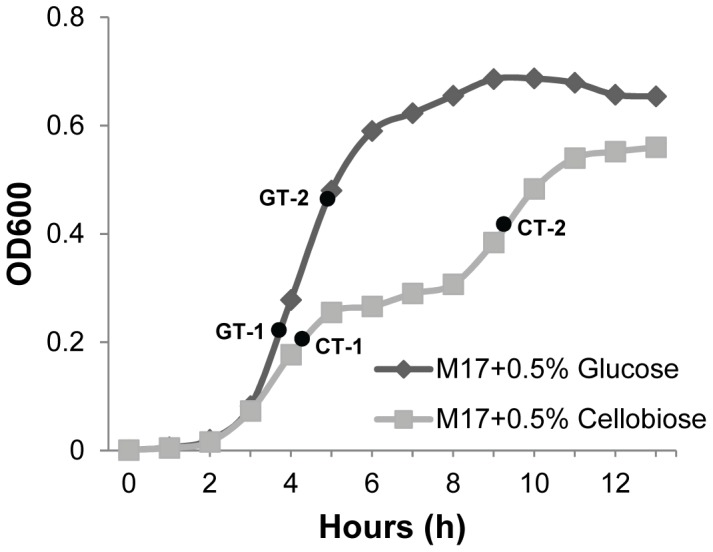
Growth of *S. pneumoniae* D39 in the presence of 0.5% cellobiose (grey line ▪) and 0.5% glucose (black line ♦) in M17 medium. Black circles show the time points at which cultures were harvested for transcriptome analysis. Where C = cellobiose, G = Glucose, T1 = time point 1 and T2 = Time point 2.

For transcriptome analyses of *S. pneumoniae*, the D39 wild-type strain was grown in 3 biological replicates in CM17 (0.5% Cellobiose+M17) and compared to the strain grown in GM17 (0.5% Glucose+M17) medium. Cells were harvested for RNA isolation at two time points in CM17 (CT-1 and CT2) and GM17 (GT-1 and GT-2) medium (see Fig. 1). To analyze the effect of *bguR* deletion on the transcriptome, *S. pneumoniae* D39 wild-type and its isogenic *bguR* mutant (SS300) were grown in 3 biological replicates in GM17 (0.5% Glucose+M17) medium. These cultures were harvested at the mid-exponential phase of growth at an OD600 of 0.25. All other procedures regarding the DNA microarray experiments (cell disruption, RNA isolation, RNA quality testing, cDNA synthesis, labeling with dyes (Cy3 and Cy5), hybridization and scanning) were performed as described before [27], [28].

### DNA Microarray Data Analysis

DNA microarray data were analyzed as described before [27]–[29]. For identification of differentially expressed genes a Bayesian p-value <0.001 and fold change cut-off of 3 was applied. Microarray data have been submitted to GEO under accession number GSE43345.

### *In silico* Analyses

The NCBI web site (www.ncbi.nlm.nih.gov/BLAST/) was used for blasting genes among different genomes, while the Kyoto Encyclopedia of Genes and Genomes (www.genome.jp/kegg/) database was used to analyze different genomes of *S. pneumoniae* (D39, Tigr4, R6, GSSP14, G54-19F, ATCC700669, Hungary-19A, JJA, P1031, Taiwan-19F-14, TCH8431-19A, 630-6B, 70585 and AP200) for the presence of the *cel* gene cluster and *bgu* operon. The in-house developed software Genome 2D (http://server.molgenrug.nl/) was used to analyze the whole genome of *S. pneumoniae* D39 for the presence of BguR operator sites, allowing 4 mismatches.

## Results

### Growth and Transcriptome Analysis of *S. pneumoniae* in Medium with Cellobiose as the Sole Carbon Source

To study the growth behavior of *S. pneumoniae* D39 in cellobiose, growth experiments were performed in the complex M17 medium supplemented with 0.5% cellobiose, which was compared to the growth in M17+0.5% glucose. As expected, normal exponential growth of D39 was observed in the presence of glucose. However, on cellobiose cells grew in a diauxic growth pattern with two distinct growth phases. This is similar to the growth behavior of *S. pneumoniae* on β-glucosides like cellobiose as seen in a recent study [18]. As the first exponential growth phase is similar to the growth in M17 without added carbon source (Fig. 1), it is most likely that cellobiose is only metabolized in the second growth phase. Because of these two growth phases on cellobiose, we decided to perform a transcriptome comparison with the growth on glucose in both phases (T1 and T2, Fig. 1) and in this way identify cellobiose-responsive genes. Therefore, D39 wild-type was grown in CM17 (0.5% Cellobiose+M17) and in GM17 (0.5% Glucose+M17) and cells were harvested at two different time points (T1 and T2) as indicated in Fig. 1. Table 3 summarizes the number of genes that were affected in these transcriptome profiling experiments, grouped into COG (Clusters of Orthologous Groups) functional categories on the basis of the putative functions of the corresponding proteins (see table S1 for expression ratios of all genes that were significantly differentially expressed at time points T1 and T2). At T1 ∼10 times more genes were affected in the presence of cellobiose than at T2, comprising many genes of COG functional category J (translation, ribosomal structure and biogenesis). This probably reflects the fact that in the presence of cellobiose, cells have to adapt in the first phase and are therefore not growing optimally, whereas in the second phase the cellobiose is likely to be actively metabolized. At both time points (T1 and T2), most of the affected genes belong to COG functional category G (carbohydrate transport and metabolism). Almost all the affected genes belonging to category G (49 out of 51 at T1 and 16 out of 20 at T2) were upregulated in the presence of cellobiose. These effects may be either due to release of carbon catabolite repression [13] of these genes as an effect of the absence of glucose, or due to a direct inductive effect of cellobiose. Other COG functional categories with a high number of differentially expressed genes are S (function unknown) and R (general functions prediction only).

**Table 3:** Number of genes significantly* affected in the presence of cellobiose at time point T1 and T2.

Functional Categories		T1			T2	
	Total	Down	Up	Total	Down	Up
C: Energy production and conversion	7	1	6	1	0	1
D: Cell cycle control, cell division, chromosome partitioning	5	4	1	0	0	0
E: Amino acid transport and metabolism	18	16	2	0	0	0
F: Nucleotide transport and metabolism	14	12	2	0	0	0
**G: Carbohydrate transport and metabolism**	**52**	**2**	**50**	**9**	**0**	**9**
H: Coenzyme transport and metabolism	9	4	5	0	0	0
I: Lipid transport and metabolism	3	2	1	0	0	0
J: Translation, ribosomal structure and biogenesis	49	46	3	0	0	0
K: Transcription	12	7	5	1	0	1
L: Replication, recombination and repair	13	10	3	0	0	0
M: Cell wall/membrane/envelope biogenesis	15	9	6	0	0	0
O: Posttranslational modification, protein turnover, chaperones	6	4	2	3	0	3
P: Inorganic ion transport and metabolism	8	6	2	0	0	0
Q: Secondary metabolites biosynthesis, transport and catabolism	1	0	1	0	0	0
R: General function prediction only	27	13	14	2	0	2
S: Function unknown	51	22	29	10	2	8
T: Signal transduction mechanisms	3	1	2	0	0	0
U: Intracellular trafficking, secretion, and vesicular transport	2	1	1	0	0	0
V: Defense mechanisms	12	3	9	0	0	0
**Total number of genes**	**307**	**163**	**144**	**26**	**2**	**24**

* Representing the genes with at least 3-fold increase or 3-fold decrease in expression levels in CM17 compared to GM17, and with a Bayesian p-value below 0.001 (Cyber-T test).

The effects of cellobiose on the transcriptome of *S. pneumoniae* D39 that were observed at both time points are summarized in Table 4. The expression of various genes and operons of diverse function was altered in the presence of cellobiose instead of glucose as the added carbon source, among which sugar metabolic genes. Notably, genes involved in cellobiose and maltose metabolism were highly upregulated. In addition, an ABC transporter cluster (*msmEFG*) that encodes a putative multiple sugar transport system was highly upregulated as well. Cellobiose metabolism has previously been shown to be carried out by a cellobiose-specific gene cluster (*cel* locus) in streptococci, including *S. pneumoniae* [7], [8], [21]. In a previous study, we showed that CelR is involved in the activation of the *cel* locus, specifically in the presence of cellobiose [8]. As expected, the *cel* locus was also among the highly upregulated genes at both time points. This suggests that the conditions applied for the transcriptome analyses are indeed appropriate to find cellobiose-responsive genes. The expression of an operon (*SPD1830–1833*, which will be denoted here tentatively as the *bgu* operon) encoding a glycosyl hydrolase protein and the PTS system IICBA components was also highly upregulated at both time points of growth. Interestingly, blast searches showed that this operon has high similarity with the *cel* locus that was previously identified to be involved in cellobiose metabolism [7], [8]. In addition, this operon was recently shown to play a role in the metabolism of the β-glucosides cellobiose and amygdalin [17]. Our transcriptome data support a role of this *bgu* operon in the metabolism/utilization of cellobiose or similar carbon sources. Therefore, we decided to further investigate the regulation of this operon in the presence of cellobiose.

**Table 4:** List of genes that are differentially expressed in the transcriptome comparison of *S. pneumoniae* D39 strain grown in CM17 and GM17 at time points T1 and T2.

D39 locus tag^a^	Function (TIGR Annotation)^b^	Ratio^c^		
		^T1^	^T2^	
*SPD0265*	Alcohol dehydrogenase, zinc-containing	26.0		2.1
*SPD0277*	6-Phospho-beta-glucosidase, CelA	71.9		88.4
*SPD0278*	Hypothetical protein	2.1		3.5
*SPD0279*	Cellobiose phosphotransferase system IIB component, CelB	13.9		23.4
*SPD0280*	DNA binding transcriptional regulator, CelR	10.3		10.5
*SPD0281*	Cellobiose phosphotransferase system IIA component, CelC	11.7		6.9
*SPD0282*	Hypothetical protein	12.8		4.3
*SPD0283*	Cellobiose phosphotransferase system IIC component, CelD	8.8		3.7
*SPD0344*	DNA-binding response regulator	3.5		2.8
*SPD0466*	BlpT protein fusion	4.3		4.0
*SPD0473*	Immunity protein BlpY	11.8		5.6
*SPD0502*	PTS system, beta-glucosides-specific IIABC components	10.1		1.3
*SPD0503*	6-phospho-beta-glucosidase	14.6		1.1
*SPD0661*	PTS system IIABC components	2.0		2.1
*SPD0850*	Lactoylglutathione lyase, GloA	−20.6		2.5
*SPD0851*	Dihydroorotate dehydrogenase, PyrK	−150.3		2.8
*SPD0852*	Dihydroorotate dehydrogenase 1B, PyrDb	−65.8		2.3
*SPD0886*	Thioredoxin family protein	12.6		3.6
*SPD1495*	Sugar ABC transporter, sugar-binding protein	8.5		7.1
*SPD1496*	PTS system. IIBC components	2.7		2.7
*SPD1590*	General stress protein 24. putative	5.5		5.1
*SPD1675*	Sugar ABC transporter, MsmG	8.2		13
*SPD1676*	Sugar ABC transporter, MsmF	11.9		1.7
*SPD1677*	Sugar ABC transporter, MsmE	10.7		2.1
*SPD1726*	Pneumolysin, PlY	−2.0		−2.0
*SPD1727*	Hypothetical protein	−2.7		−2.5
*SPD1728*	Hypothetical protein	−3.5		−3.6
*SPD1729*	Hypothetical protein	−8.64		−3.4
*SPD1830*	Glycosyl hydrolase family 1, BguA	57.1		2.3
*SPD1831*	PTS system. IIC component, BguD	62.9		3.4
*SPD1832*	PTS system. IIB component, BguB	53.4		4.2
*SPD1833*	PTS system. IIA component, BguC	55.9		3.3
*SPD1865*	Alcohol dehydrogenase. Zinc-containing	12.6		2.5
*SPD1866*	N-Acetylglucosamine-6-phosphate deacetylase, NagA	4.2		2.5
*SPD1933*	Glycogen phosphorylase family protein	1.3		1.7
*SPD1934*	4-Alpha-glucanotransferase, MalQ	2.1		3.4
*SPD1935*	Maltose/maltodextrin ABC transporter, MalX	1.3		2.1
*SPD1936*	Maltodextrin ABC transporter, MalC	1.6		1.8

a Gene numbers refer to D39 locus tags.

b D39/TIGR4 annotation [10], [15], [47],

c Ratio represents the fold increase in the expression of genes in CM17 as compared to GM17. In some cases neighbouring genes with lower than 3-fold ratios are also indicated.

### Organization and Conservation of the *bgu* Operon in *S. pneumoniae*

**Figure 2:**
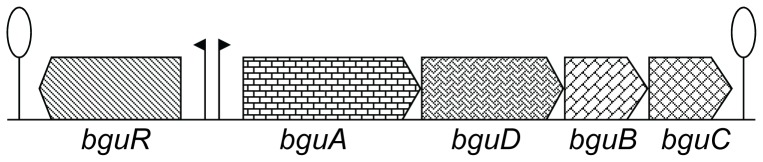
Organization of the *bgu* operon in *S. pneumoniae* D39. Lollipop structure represents transcriptional terminator. Black arrows represent promoter regions. See text for further details.

The *bgu* locus spans the genes *SPD1830-33* (Fig. 2). *SPD1830* (here named *bguA*) encodes a glycosyl hydrolase belonging to the BglB family, which has high similarity (30% identity) to *celA* of *S. pneumoniae* and other streptococci. Next, the downstream three genes (*SPD1831-33*, named *bguDBC*), encode PTS system IICBA components that show high similarity (29–30% identity) with *celDCB* located in the *cel* locus of various streptococci, including *S. pneumoniae*. Upstream of *bguA*, a gene encoding a GntR family transcriptional factor (named *bguR*) is located. The presence of this transcription factor in the DNA region upstream of the *bgu* operon indicates that it may function as a transcriptional regulator of the *bgu* operon (see also below).

In a previous study, we have studied the role of CelR in the regulation of the *cel* locus [8]. However, the *cel* locus was found to be absent from 50% of the *S. pneumoniae* strains present in the KEGG database. We also analyzed all the strains of *S. pneumoniae* available on the KEGG website for the presence of the *bgu* operon. Notably, this operon is 100% conserved in all the sequenced strains of *S. pneumoniae* available on KEGG database. This suggests an important role of this operon in carbohydrate metabolism, probably of cellobiose or related/analogous compounds [17]. In subsequent experiments, we focused in more detail on the molecular basis of the transcriptional regulation of the *bgu* operon.

### Cellobiose-dependent Expression of the *bgu* Operon

To examine whether the observed altered expression of the *bgu* operon in the transcriptome analyses was due to a specific effect of cellobiose, we constructed an ectopic transcriptional *lacZ*-fusion to the *bguA* promoter in the D39 wild-type strain. This strain was grown in M17 medium supplemented with 0.5% of different carbon sources (Cellobiose, Fructose, Galactose, Glucose, Lactose, Mannose, Maltose, NAG, Raffinose, Sucrose and Trehalose). The highest expression of P*bguA* was observed in the presence of cellobiose (Table 5). The presence of glucose led to the lowest expression of P*bguA*. These results confirm the microarray data and additionally show that expression of P*bguA* is controlled specifically by cellobiose.

**Table 5:** Specific β-galactosidase activity (miller units) of D39 wild-type containing the P*bguA-lacZ* fusion grown in M17 medium with different added sugars (0.5% w/v).

Specific β-Galactosidase Activity (Miller Units) in M17 medium	
Sugars	P*bguA-lacZ*
No	124 (4)
Cellobiose	515 (6)
Fructose	121 (9)
Galactose	122 (7)
Glucose	105 (6)
Lactose	125 (9)
Mannose	129 (5)
Maltose	131 (6)
NAG	130 (8)
Rafinose	116 (7)
Sucrose	103 (5)
Trehalose	125 (4)

Standard deviation of 3 independent experiments is given in parentheses.

To further investigate the role of glucose in the regulation of P*bguA*, we grew the cells in the presence of a certain constant concentration of glucose, with an increasing concentration of cellobiose, as mentioned in Table 6. At 0.2% glucose, the lowest expression of P*bguA* was observed. This level of expression could not be increased by adding 0.5% cellobiose to the medium. Thus, cellobiose increases the expression of P*bguA*, whereas glucose decreases its expression, whereby the last compound appears to overrule the first when present at the same time.

**Table 6:** Specific β-galactosidase activity (miller units) of D39 wild-type containing the P*bguA-lacZ* fusion grown in M17 medium with different combinations of added sugars (% w/v).

Specific β-Galactosidase Activity (Miller Units) in M17 medium	
Sugars	P*bguA-lacZ*
No	115 (2)
0.1% C	205 (3)
0.2% C	340 (5)
0.3% C	380 (5)
0.5% C	490 (17)
1% C	650 (13)
0.1% G	109 (2)
0.2% G	107 (3)
0.3% G	103 (7)
0.5% G	105 (5)
1% G	101 (8)
0.1% G +0.1% C	112 (6)
0.1% G +0.2% C	122 (4)
0.1% G +0.5% C	140 (8)
0.2% G +0.1% C	109 (1)
0.2% G +0.2% C	108 (3)
0.2% G +0.5% C	112 (5)

Standard deviation of 3 independent experiments is given in parentheses. G, Glucose. C, Cellobiose.

### *SPD1829* (*bguR*) is a Repressor of the *bgu* Operon

Adjacent to the *bgu* operon, a putative GntR-family regulator is located (*SPD1829*), which we named BguR. We hypothesized that this regulator could be involved in the observed regulation of the *bgu* operon. To investigate the role of BguR, the *bguR* gene was replaced by a spectinomycin-resistance marker by means of allelic replacement. To examine the effect of the *bguR* deletion on the transcriptome of *S. pneumoniae*, the transcriptome of the *bguR* mutant strain (SS300) was compared to that of the D39 wild-type strain grown in GM17 (0.5% glucose+M17) medium. GM17 medium was used, since low expression of the *bgu* operon was observed in the presence of glucose, which we hypothesized to represent a condition with maximal repression of the *bgu* operon by BguR. These transcriptome data revealed that the expression of 13 genes was significantly altered due the deletion of *bguR* (Table 7). The expression of *bguR* was five-fold downregulated confirming the inactivation of the *bguR* gene in the *bguR* deletion strain (SS300). The most highly upregulated genes were the ones constituting the *bgu* operon (more than 20-fold), indicating a role/function of BguR as the repressor of the *bgu* operon. Another strong effect caused by deletion of *bguR* was the upregulation of an operon involved in maltose/maltodextrin metabolism [30]. These results show that inactivation of *bguR* brings about only a modest change in the transcriptome of *S. pneumoniae* D39, and furthermore imply a role of BguR in repressing the expression of the *bgu* operon.

**Table 7:** Summary of transcriptome comparison of *S. pneumoniae* strain D39 Δ*bguR* and D39 wild-type grown in GM17.

D39 locus tag^a^	Function^b^	Ratio^c^
*SPD0311*	Glucan 1,6-alpha-glucosidase, DexB	3.2
*SPD0771*	Lactose phosphotransferase system repressor, LacR	4.3
*SPD0772*	1-phosphofructokinase, putative	4.1
*SPD0773*	PTS system, fructose specific IIABC components	4.0
*SPD1830*	Glycosyl hydrolase, family 1, BguA	89.6
*SPD1831*	PTS system, IIC component, BguD	127.8
*SPD1832*	PTS system, IIB component, BguB	47.4
*SPD1833*	PTS system, IIA component, BguC	21.8
*SPD1932*	Glycogen phosphorylase family protein	10.0
*SPD1933*	4-alpha-glucanotransferase, MalQ	14.0
*SPD1934*	Maltose/maltodextrin ABC transporter, MalX	4.7
*SPD1935*	Maltodextrin ABC transporter, MalC	2.6
*SPD1829*	GntR family transcriptional regulator, BguR	−5.2

a Gene numbers refer to D39 locus tags.

b D39 annotation/TIGR4 annotation [10], [15], [47],

c Ratio represents the fold increase in the expression of genes in CM17 as compared to GM17.

### Regulation of P*bguA* in *ccpA*, *bguR* and *bguDBC* Mutant Strains

To further confirm that BguR is responsible for the repression of the *bgu* operon that was upregulated in the *bguR* mutant strain (SS300), we introduced the P*bguA-lacZ* into D39 wild-type and the *bguR* mutant. In GM17 (0.5% Glucose+M17) medium specific β-galactosidase activity was highly increased in the *bguR* mutant as compared to D39 wild-type (Table 8). This not only confirms the data of the *bguR* mutant transcriptome but also shows that BguR carries out repression of the *bgu* operon via the P*bguA*.

**Table 8:** Specific β-Galactosidase activity (miller units) of D39 wild-type, Δ*bguR*, Δ*ccpA*, and Δ*bguDBC* mutants all containing the P*bguA-lacZ* transcriptional fusion grown in M17 medium supplemented with added concentrations (0.5% w/v) of cellobiose (C) and glucose (G).

Specific β-Galactosidase Activity (Miller Units)				
	*WT*	Δ*bguR*	Δ*ccpA*	Δ*bguDBC*
**P** ***bguA*** ** (GM17)**	115 (5)	1660 (17)	111 (8)	116 (9)
**P** ***bguA*** ** (CM17)**	610 (10)	1690 (15)	580 (12)	599 (17)

Standard deviation of three independent measurements is given in parentheses.

CcpA is considered a master transcriptional regulator in the control of carbohydrate utilization and metabolism genes in Gram-positive bacteria including *S. pneumoniae* [13], [31], [32]. To investigate a possible role of CcpA in the regulation of the *bgu* operon, we measured specific β-galactosidase activity of P*bguA-lacZ* in a *ccpA* mutant strain. No difference in expression of P*bguA* was observed in the *ccpA* mutant as compared to the wild-type strain when cells were grown in M17 with either cellobiose or glucose (Table 8). In addition, no CcpA binding site (*cre*) was found in the P*bguA* promoter [13]. Thus, these data show that regulation of the *bgu* operon in *S. pneumoniae* is independent of CcpA.

In *S. mutans*, regulation of the *cel* locus by CelR requires the phosphorylation of CelR by one of the PTS components, namely CelD [21]. Therefore, to investigate a possible role of the BguDBC proteins in the regulation of the *bgu* operon via an effect on BguR activity, we measured specific β-galactosidase activity of P*bguA-lacZ* in a *bguDBC* mutant strain. No difference in expression of P*bguA* was observed in the *bguDBC* mutant as compared to the wild-type D39 strain, when cells were grown in M17 medium with cellobiose or glucose (Table 8). This suggests that the components of the Bgu PTS system are not required for activation of BguR in the presence of cellobiose. However, we cannot exclude that other PTS systems or signaling cascades confer a regulatory effect on BguR.

### Identification of the BguR Operator Site

**Figure 3:**
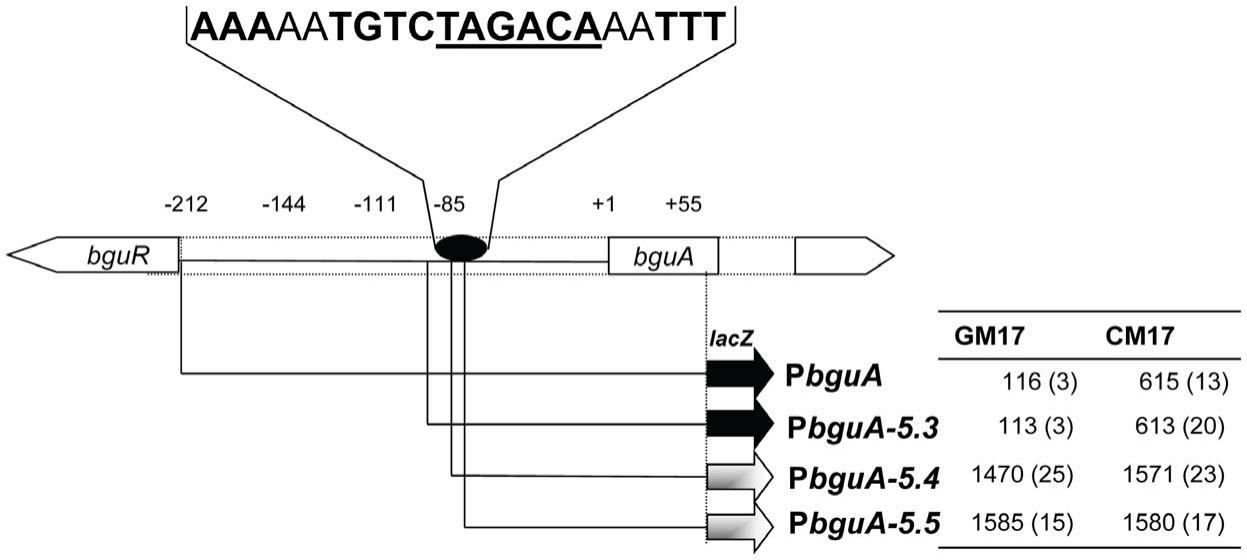
Analysis of truncations of P*bguA*. A schematic overview of the *bguA* promoter truncations is shown. The table on the right gives the specific β-galactosidase activity of the truncated promoters in GM17 (0.5% glucose+M17) and CM17 (0.5% cellobiose+M17). Standard deviation is given in parentheses. The oval indicates the position of the putative BguR operator site, while the sequence of the BguR operator site is given above.

The data presented above strongly suggest a direct effect of BguR on P*bguA*. To identify a possible BguR operator sequence, a 5′ promoter truncation study was performed with the *bguA* promoter. A diagram of the *bguA* promoter truncation is shown in Fig. 3. Truncation of P*bguA* near to the predicted -35 core promoter sequence (P*bguA-5.4*) relieved the repressive action of BguR on P*bguA,* suggesting the presence of a putative BguR operator in this deleted region of the promoter. Further bioinformatics analysis of this area revealed the presence of a 20-bp palindromic region (5′-AAAAATGTCTAGACAAATTT-3′) that is overlapping with the -35 site and that might act as the BguR operator site. Deletion of half of this predicted operator site (P*bguA-5.5*) led to high expression of P*bguA* in CM17 (0.5% Cellobiose+M17) and GM17 (0.5% Glucose+M17) medium in the wild-type (Fig. 3). However, when the P*bguA* was truncated only a few base pairs upstream of the predicted operator site (P*bguA-5.3*), expression was similar to that of the full-length promoter (Fig. 3). Therefore, these data suggest that the predicted operator site is functional and acts as the BguR operator site.

The entire genome of *S. pneumoniae* D39 was searched for the presence of the BguR operator site by using Genome2D. Also, the promoters of the putative fructose and maltose utilization gene clusters were analyzed manually for the presence of the BguR operator site. However, we could not find a sequence that resembles it, suggesting that the *bgu* operon is the only direct target of BguR.

### The *cel* Locus is Required for D39 to Grow on Cellobiose, While the *bgu* Operon is Not

**Figure 4:**
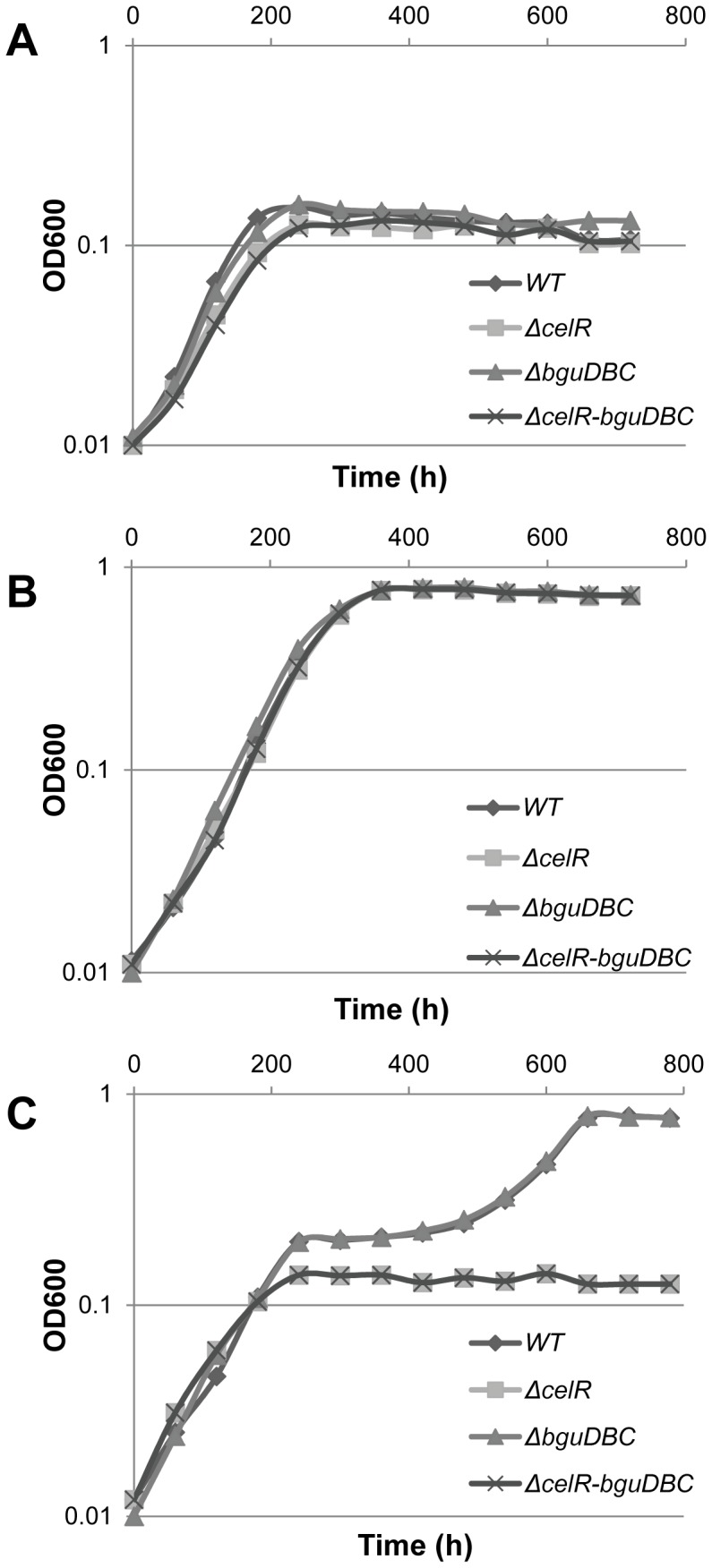
Growth of *S. pneumoniae* D39 wild-type (♦) and its isogenic mutants *celR* (▪), *bguDBC* (▴) and *celR-bguDBC* (X) in M17 (A), 0.5% glucose+M17 (B) and 0.5% cellobiose+M17 (C).

As in a previous study it had been shown that deletion of *celR* or *celD* from the *cel* locus leads to growth inhibition in CDM with cellobiose as the carbon source [7], and since the *bgu* locus was implicated in the uptake of cellobiose and amygdalin [17], we decided to mutate *bguDBC* and compare the growth of Δ*bguDBC*, Δ*celR* (which has no expression of the *cel* locus) [7], [8], and Δ*celR-bguDBC* in the presence of 0.5% cellobiose and 0.5% glucose with that of D39 wild-type (Fig. 4). In the presence of glucose, all strains grew similar as the wild-type. However, clear growth differences were observed in the presence of cellobiose. As mentioned before, D39 wild-type grows with two distinct exponential phases in the presence of cellobiose. A similar growth pattern was observed for the Δ*bguDBC* mutant strain, but the Δ*celR* and Δ*celR-bguDBC* mutant strains were not able to start the second exponential phase. These data suggest that the *cel* locus is important for *S. pneumoniae* D39 to grow on cellobiose, while under these conditions the *bgu* operon, although its expression is responsive to cellobiose, is not.

## Discussion

In many bacteria, including *S. pneumoniae*, glucose is considered one of the primary sources of energy for metabolic processes. However, the existence of numerous other sugar-specific systems in *S. pneumoniae* implies its ability to utilize various other carbon sources in the absence of glucose [10], [15]–[17]. The role of different systems dedicated to the uptake and metabolism of different sugars including cellobiose, maltose, galactose, sucrose and raffinose has been explicitly investigated for *S. pneumoniae* [7], [8], [11], [13], [16], [17], [30], [33]–[36]. In a previous study, we described the role of the cellobiose-dependent transcriptional activator (CelR) in the regulation of the cellobiose utilization gene cluster (*cel* locus) [8]. Intriguingly, the *cel* locus is not conserved in all the sequenced strains of *S. pneumoniae* that are available on the KEGG website. This might indicate that *S. pneumoniae* harbors other ways of cellobiose utilization. To be able to more thoroughly understand the effect of cellobiose on *S. pneumoniae*, we applied transcriptome profiling and various ways to study transcriptional gene regulation. The data show that, besides the *cel* locus, expression of a second operon (*bgu*) is responsive to cellobiose as well, which we show to be mediated by the transcriptional regulator BguR. The fact that the *bgu* operon is 100% conserved in different pneumococcal strains suggests that it has an important physiological function in the life style of *S. pneumoniae*. Indeed, like *celR*, *bguD* was also found in one of the STM (Signature-tagged mutagenesis) studies, where it was implicated in lung infection [37].

The phosphoenolpyruvate-dependent (PEP) phosphotransferase system (PTS) is a major carbohydrate uptake system in bacteria, which not only phosphorylates different carbohydrates during uptake but also plays a major role in genetic regulation of metabolic activities [38]–[40]. In *S. mutans*, it has been shown that CelR has two PRD (PTS regulation domain) domains and phosphorylation (by glucose and cellobiose PTSs) at H226, H332 and H576 is required for the activation of CelR in the presence of cellobiose, while phosphorylation of H284 and H391 leads to inhibition of the CelR-dependent activation in the presence of glucose [21]. A similar role of these histidine residues as well as a phosphorylatable cysteine residue located in the EIIB domain of CelR is also elaborated on in *S. pneumoniae* [18]. The phosphorylatable cysteine was shown to affect the length of the lag phase during the shift from growth on glucose to that on cellobiose and other β-glucosides. In addition, PTS SPD0502, which might transport β-glucosides as well [18], was found to be responsible for the extended lag phase, whereas the Bgu PTS that we describe here did not. Interestingly, the gene encoding the SPD0502 PTS was found to be upregulated during growth on cellobiose at T1 in our microarray experiment described in this study (Table 4 and S1). Thus, complex regulatory interactions around CelR take place.

In the BguR amino acid sequence no similar regulatory domains could be identified. In agreement with this, we could not find an effect of deletion of the *bgu* PTS genes on the cellobiose- and glucose-dependent regulation of P*bguA* via BguR. Therefore, it remains to be determined how the signal of the carbohydrate source is transferred to BguR. In addition, no effect of CcpA on the regulation of the *bgu* operon could be detected, in accordance with previous studies [13]. Thus, expression of the *bgu* locus seems to be governed by a single transcriptional regulator, BguR, possible via direct binding of glucose and cellobiose to its C-terminal UTRA (UbiC transcription regulator-associated) domain and the regulation of the *bgu* locus is independent of the Cel system.

As reported in our previous study, the *cel* locus and the CelR regulatory site of *S. pneumoniae* were found to be highly conserved in other streptococci, although not all pneumococcal strains contain this operon [7]. Blast searches revealed a high conservation of the *bgu* operon in all the strains of *S. pneumoniae* that are available on the KEGG website. However, no similar *bgu* operon organization could be found in other streptococcal species using BLAST searches. Thus, the *bgu* operon seems to be specific for *S. pneumoniae*.

Blast searches with BguR revealed high similarity of this protein with the previously characterized GntR-type transcriptional regulator GmuR in *Bacillus subtilis* [41], BgcR in *Escherichia coli* [42] and DasR in *Streptomyces coelicolor* [43]. Moreover, the BguR operator site that we proposed in this study is also similar to the predicted GmuR (5′-tAAATGTaTAGACAttTa-3′) operator site in the order of bacillales [44]. GmuR was found to be involved in the regulation of the *gmuBACDREFG* operon, encoding glucomannan utilization genes [41]. Expression of this operon was induced by cellobiose and mannobiose, which are possible degradation products of the action of GmuG on glucomannan, and repressed by glucose [41]. Interestingly, GmuBAC has 31–46% identity with BguDBC while GmuR has 46% identity with BguR. Although we did not observe an effect of deletion of the *bgu* operon on growth on cellobiose, it could be involved in the utilization of other glucomannan degradation products such as 4-O-β-D-mannopyranosyl-D-mannopyranose (β-1,4-D-mannobiose), and 4-O-β-D-glucopyranosyl-D-mannopyranose. Intestinal anaerobic bacteria encounter glucomannan and are able to degrade and ferment it [45]. Therefore, it is possible that the degradation products of glucomannan are present in the environment of *S. pneumoniae* as well, by uptake from the intestine.

BgcR is a positive transcriptional regulator of a *bgc* operon which consists of 5 genes including genes encoding PTS subunits IIB (BgcE), IIA (BgcF), IIC (BgcI) and phospho-β-glucosidase (BgcA), and is found to be involved in the utilization of cellobiose and other β-glucosides (arbutin and salicin) at low temperature in *E. coli* [42]. However, salicin was not found to be fermented by the Bgu system and arbutin was shown to be taken up by the Cel system in *S. pneumoniae* [7], [17]. Given the high similarity of the Bgc proteins with the proteins encoded in the *bgu* operon, we cannot exclude a role of the *bgu* operon in the utilization of arbutin or analogous sugars that *S. pneumoniae* encounters in its natural environment.

Deletion of the genes encoding the PTS components (*bguDBC*) of the *bgu* operon has no effect on growth compared to the wild-type in the presence of cellobiose as the sole carbon source, whereas deletion of *celR* (impairing expression of the *cel* locus) does. Recent studies in *S. pneumoniae* R6 have shown that deletion of *bguD* did lead to an increased lag-phase on cellobiose and a slight effect on growth on amygdalin [17], [18]. However, deletion of *celD* led to strong inhibition of growth of *S. pneumoniae* R6 on amygdalin, cellobiose, esculin and gentiobiose [17]. Thus, the *cel* locus is primarily responsible for β-glucoside utilization in *S. pneumoniae* and the exact role of the *bgu* operon in the uptake and metabolism of β-glucosides remains to be determined.

Interestingly, the BguR operator site could only be found in the promoter region of the *bguA* gene. This suggests that the *bgu* operon is the only direct target of BguR. However, deletion of *bguR* also led to upregulation of maltose- and fructose utilization gene clusters. The *mal* gene cluster is regulated by *malR*, a maltose-dependent transcriptional repressor [30], [46]. Therefore, the upregulation of the *mal* gene cluster might be an indirect effect of the high upregulation of the *bgu* operon. However, the regulatory mechanism of the putative fructose utilization gene cluster in *S. pneumoniae* has not yet been explored. It could well be that the LacR type regulator encoded in this gene cluster directly regulates it.

## Supporting Information

Table S1 Overview of all significantly up- or downregulated in the presence of cellobiose, compared to growth in the presence of glucose, at time point T1 and T2.
(XLSX)
